# PROTOCOL: School‐based education programmes for improving knowledge of back health, ergonomics and postural behaviour of school children aged 4–18: A systematic review

**DOI:** 10.1002/cl2.1014

**Published:** 2019-07-13

**Authors:** Josette Bettany‐Saltikov, Robert McSherry, Paul van Schaik, Gokulakannan Kandasamy, Julie Hogg, Vicki Whittaker, Garikoitz Aristegui Racero, Tracey Arnell

**Affiliations:** ^1^ School of Health and Social Care Teesside University Middlesbrough UK; ^2^ School of Social Sciences, Humanities and law Teesside University Middlesbrough UK; ^3^ School of Health Basque University San Sebastián Spain

The health of school aged children's backs is a very important topic worldwide. Many schools require children to sit for long periods of time and the increasing use of technology adds to the number of hours they are seated. As back pain is increasingly being reported in young people, an understanding of spinal health and posture is essential for students, teachers and parents. The causes of back pain in young people are challenging and wide ranging. These include; mechanical back shape and incorrect posture while using technology (Game Consoles, Computers, Gameboys, iPads and iPhones). Furthermore, carrying heavy back packs, improper lifting techniques, incorrect posture during prolonged sitting and standing, together with a lack of back care knowledge and the opportunity to move around frequently (in standing, sitting) can lead to poor back health. These issues may be made worse by the school environment, for example, the unavailability or inadequate numbers of school lockers, ill‐fitting school furniture and the changing routine of the school day which don't allow children to move around as much as they would like to.

Back health educational programmes aim to decrease the possibility of spinal, back and other problems which may lead to disability and pain in muscles and bones. The aims of these programmes are varied and diverse and have included numerical, physical, mechanical, positional, environmental and even social factors. Numerical as in trying to decrease the numbers of students with back pain. Mechanical as in improving body mechanics, posture and safety; as well as methods of wearing a backpack. Positional associated with teaching good sitting postures, safe lifting techniques, sports injury prevention procedures, as well as training students to make appropriate and safe decisions regarding the use of their bodies. These are essential in order to prevent the onset of back pain as well as improving students’, teachers and parents knowledge of back care principles.

Further research has shown that “hands‐on” learning or learning by doing is much more effective than just being spoken to in a classroom. As research in this area is still very unclear, a systematic review conducted with state of the art, high‐quality Campbell Collaboration methodology is urgently needed to update parents, children, teachers, researchers and clinicians with the latest research evidence to help educate/inform everyone involved in this issue and also to inform changes in policy and practice in this area of public health.

## 1 BACKGROUND

### 1.1 Description of the condition

#### 1.1.1 The problem, condition or issue

##### Description of the condition

Musculoskeletal and back Health of school aged children is a global health problem with evidence that the prevalence of these problems is increasing (Rajan & Koti, 2013; Yao, Mai, Luo, Ai, & Chen, 2011). As Duggan states “many modern school systems require children to assume sedentary positions for extended periods of time, and the increasing use of classroom‐based technology adds to the number of hours seated. With the incidence of musculoskeletal pain reported not only in adult populations but increasingly in young people, an understanding of spinal health and posture may be essential for students”. Posture is the attitude assumed by the body either when it is stationary or when it is moving. It is attained because of the coordinated action of various muscles working to maintain stability (Gardiner, 1957). Poor posture is the result of musculoskeletal distortion in the neck and lower and upper back. Most people think of poor posture as simply slumping over, but this is not necessarily the case. Further due to the variety of body types, incorrect posture differs from person to person. One person's proper posture can be an incorrect posture for someone else and vice versa. Posture holds the body upright against gravity while standing, sitting or lying down. The ideal “normal” erect posture is one in which the line of gravity (the vertical line drawn through the body's centre of gravity) runs when viewed from each side. In layman's terms, this means that good posture (Kisner, Colby & Borstad, 2017) is the position which is attained when the joints are not bent or twisted, and the spine is aligned. Maintaining good posture involves training one's body to move and function where the least strain is placed on the bones, joints and soft tissues. Poor posture can result in numerous health problems such as tight neck muscles, muscle soreness, pain (shoulder, neck, back and arms), headaches, poor circulation, physical and mental stress as well as poor sleep.

To prevent the health problems mentioned above, it is important to “fit” any work or school environment to the user's needs (correct ergonomics; Rajan & Koti, 2013). Ergonomics aims to increase efficiency and productivity by reducing discomfort. A sound understanding of ergonomics by students can help prevent school place injuries by adjusting the tools (e.g., desk, chair, computer screen) to the user, putting an emphasis on proper posture to reduce the impact of repetitive movements or the potential for straining muscles and joints. The use of computers together with the rapidly changing technology in modern schools has greatly increased the need for ergonomics. Desks, chairs, monitors, keyboards and lighting all need to be assessed when creating a work or school space, whether it is at the office, at home or at school. It is important to study ergonomics because faulty ergonomics has been known to cause musculoskeletal aches and pains (Sellschop, Myezwa, Mudzi, & Musenge, 2018). Ergonomic assessment, especially in schoolchildren, is gaining ground as the activities and the postures used to perform those activities could be one of the reasons for the high prevalence of pain in this young population, which may lead to chronic pain in adulthood (Rajan & Koti, 2013; Yao et al., 2011).

The International Association for the Study of Pain defines pain as “an unpleasant sensory and emotional experience associated with real or potential tissue injury or described as caused by this injury or whose presence is revealed by visible and/or audible behaviour manifestations” (Merskey & Bogduk, 1994). Recent population‐based studies conducted in a range of countries have reported point, 1‐year and lifetime prevalence rates of back pain in adult populations of up to 52%, 84% and 91%, respectively (Steele, Dawson, & Hiller, 2006). However, spinal pain is an issue across the lifespan, not just in adulthood. Cross‐sectional surveys conducted in different countries with children and adolescents between the ages of 8 and 16 years of age variably report 1‐month prevalence rates of up to 39%, and lifetime prevalence rates of back pain up to 69.3% (Kovacs et al., 2003; Watson et al., 2002; Wedderkopp, Leboeuf‐Yde, Andersen, Froberg, & Hansen, 2001). Although posture as well as movement may not appear to be directly relevant in the context of schooling, in western Europe most courses are either dedicated to improving the brain or the body (Dugan, 2018). Within school settings, the latter are usually considered to be secondary to the former. As Dugan (2018) states “There is, evidence to suggest that the body and brain are inextricably linked and that physical health is valuable if not essential for educational pursuits; this can be demonstrated by links between body and memory retrieval (e.g., Dijkstra, Kaschak, & Zwaan, 2007), logical concept acquisition (Fischer & Brugger, 2011) as well as emotional and personality factors (e.g., Pitterman & Nowicki, 2004), all of which come into play in the classroom”.

The causes of back pain in youth are challenging and difficult to diagnose and as briefly mentioned above have been theorised to include; mechanical back shape, incorrect posture while using technology (game consoles, computers, Gameboys, iPad and iPhone) and carrying heavy back packs, improper lifting techniques, incorrect posture during prolonged sitting and standing, together with a lack of back care knowledge and the lack of opportunity to move around frequently (in standing, sitting). These issues may be exacerbated by the educational environment because of inadequate or unavailable school lockers, ill‐fitting school furniture and the changing structure of the school day which provides fewer opportunities for movement (Bettany‐Saltikov, Warren, & Stamp, 2008; Cardon, Dirk, Ilse, & Dieter, 2004; Feingold & Jacobs, 2002; Legg & Cruz, 2004; Sheldon, 1994). Dugan (2018) further suggests that because of the significance of the human body in learning, studies have looked at all the above causes that could potentially impact how students' bodies function in educational settings. Interventions have also been conducted helping students discriminate between healthy and poor postures, reminding them to sit properly at their desks and computer workstations and modifying their physical habits and routines. Historically, educating children and youth about the importance of back health and posture was an important element of the physical education national curriculum in many countries but was overlooked during the 1980s when heart health became a greater priority (Tinning, 2001). The high prevalence rates of back pain in children and adolescents, as well as the predictive value of adolescent spinal pain for spinal pain in adults, have caused several authors to advocate for, and implement, spinal health interventions in the school setting (Miñana‐Signes, Manuel, & Samuel, 2019).

Cardon et al. (2004) further suggest that guidelines to support teachers made a difference to the back health of school children and strongly recommend that guidelines are formulated. Back care knowledge among children, parents and teachers has also been reported to be very poor (Arghavani, Zamanian, Ghanbary, & Hassanzadeh, 2014). This is further compounded by the lack of media coverage and governmental structured programmes to help to inform youth (Bettany‐Saltikov et al., 2008; Cardon et al., 2004). This has subsequently resulted in not only children but also parents and teachers being unaware of the prevalence and risk factors of musculoskeletal pain and disorders (Arghavani et al., 2014; Habybabady et al., 2012) resulting in the increase of poor back health behaviours routinely being undertaken in schools (Salminen, Erkintalo, Laine, & Pentti, 1995). Numerous authors have also suggested that poor posture, ergonomics and body use in childhood may cause a further increase in these issues in future years (Harreby, Kjer, Hesselsøe, & Neergaard, 1996). It is generally assumed that as “young people are more flexible and adaptive learners than adults this may make childhood the best time to effect postural change and undo the physical harm modern lifestyles can inflict while at the same time setting up young people for healthy body use as adults. If this is true to any degree, elementary schools may be the best opportunity to introduce important concepts about healthy posture to learners” (Dugan, 2018, p. 643). To summarise, the patterns, disciplines and habits, whether correct or incorrect, that are learnt during children's school years have an influence on the possible development of back pathology in the future. Many authors consider that the appearance and development of back pain among teenagers is a risk factor for back pain in adults (Harreby et al., 1996) with the risk increasing the more pain one has suffered during adolescence (Hestbaek, Leboeuf‐Yde, & Kyvik, 2006; Hestbaek, Leboeuf‐Yde, Kyvik, & Manniche, 2006).

### 1.2 Description of the intervention

School environment interventions are strongly supported by the World Health Organisation framework for health promoting schools (Barnekow et al., 2006). Indeed school‐based education interventions for improving knowledge of back health and postural behaviour have been used in numerous countries worldwide to improve children's and adolescents' knowledge of back health, postural awareness and behaviour (Dugan, 2018). Back health educational programmes aim to decrease the risk of spinal, back and other musculoskeletal problems which may lead to disability and musculoskeletal pain both in the present and in the future (Arghavani et al., 2014; Habybabady et al., 2012; Vidal et al., 2013). Whilst numerous school‐based education programmes have been implemented to reduce smoking, decrease alcohol consumption and teenage pregnancies, increase physical activity and healthy eating, and prevent obesity in children and youth, educational programmes teaching children and youth about the importance of back health, posture and ergonomics, together with ways of preventing back pain, have not received as much attention. There is currently no standard school‐based educational programme for improving knowledge of back health, ergonomics and postural behaviour of school children. These programmes are designed to support students' academic success in educational establishments. Educational establishments, or schools are broadly defined as institutions dedicated to education. These interventions generally engage school children in some form of active learning that cognitively and physically engages them in learning to improve knowledge, ergonomics and postural behaviour (Dugan, 2018). The programmes described in the literature vary from country to country as well as within countries. The contents of the education back health programmes have included lectures or lectures with actual demonstrations, practical sessions, workshops, individual lessons, class group lessons, curriculum lessons, posters, hands on learning as well as educational modules. These studies have varied in their aims, the teachers teaching the intervention, the duration and intensity of the interventions as well as the content and strategies of the programmes. These are discussed in turn below:

#### 1.2.1 Aims of the educational programmes

The aims of the programmes are varied and have included all the following: to decrease the prevalence of back pain, to improve body mechanics and improve posture while performing various tasks, to improve the safety as well as methods of wearing a backpack, to teach acceptable sitting postures, safe lifting techniques and sports injury prevention procedures, to train students to make appropriate and safe decisions regarding the use of their bodies in order to prevent the onset of back pain as well as to improve students' knowledge of back care principles Steele et al. (2006).

#### 1.2.2 Teachers of back health programmes

Steele et al. (2006) systematic review on school‐based interventions for spinal pain have reported that the teachers of these back‐health education programmes are diverse and have included all of the following: the classroom teacher, physical therapists, occupational therapists, physical education teachers as well as physical therapy students.

#### 1.2.3 Duration and intensity of these programmes

The duration and intensity of these programmes have varied widely: they range from one 30 min session, one 60 min session, six 1 hr sessions over 6 weeks, one session with an unspecified duration, 11 sessions over 8 weeks (total 19 hr), three sessions of unspecified duration, and 3 years duration with no intensity specified (Steele et al., 2006).

#### 1.2.4 Content of the back‐health programmes

The reported content of these programmes (Steele et al., 2006) have included the following: principles of Swedish back school, anatomy, physiology, spinal care principles and exercises, how to choose, lift and wear backpacks safely, how to recognise when a backpack is too heavy, exercises combined with behavioural intention and self‐monitoring, biomechanics and risk factors for injury as well as how to incorporate this knowledge into everyday life using lifting techniques.

#### 1.2.5 Strategies for the delivery of these programmes

The strategies for the delivery of these programmes have included a variety of approaches. Cardon et al.'s (2004) back health programme has included teacher and parent involvement, posters in the classroom or have been included within the curriculum where guided self‐discovery and active hands‐on methods were used. Goodgold and Nielsen (2003) used a whole school approach with teacher and parent involvement, posters in the classroom and inclusion in the curriculum of the following: lecture, worksheets, demonstration, hands‐on activity. In the Goodgold and Nielsen, (2003) study, the strategies for the delivery of these programmes were altered slightly to cater to a younger age group. Mendez and Gómez‐Conesa, (2001) used teacher and parent involvement whilst also including posters in the classroom. The programme was included in the curriculum as lectures, demonstrations and practice. Robertson and Lee (1990) included the information in the curriculum only and comprised of lectures, worksheets, games, demonstrations and practice whilst Schwartz and Jacobs, (1992) strategy was included in the curriculum only: and comprised a lecture, demonstration and practice. Sheldon's (1994) strategy was also only included in the curriculum and comprised a lecture and demonstration practice and lastly, Spence et al. (1984) strategy was also only included within the curriculum and comprised a lecture, demonstration or guided self‐discovery

### 1.3 How the intervention might work

Many schools have traditionally held a “transmissionist” or “instructionist” model in which a teacher or lecturer “transmits” information to pupils, for instance giving a lecture or presentation. In contrast, Vygotsky's theory promotes learning contexts in which pupils play an active role in learning. The roles of the teacher and pupil are therefore shifted, as a teacher collaborates with his or her pupils to help facilitate meaning construction in their pupils. Further when an educational intervention is planned for children, for optimal learning to occur, children taking part need to have a greater reliance on so‐called “hands‐on” learning, or learning by doing (Hartman, Kopp, & Nelson, 2000). Hartman et al. (2000) focused on the effect of learning by doing versus learning by demonstration. These authors stated that children who had participated in a “hands‐on” project had a significantly greater amount of recall of the task than children who only had a demonstration. The children who were taught in the “hands‐on” condition also had a greater memory recall of the process. The results of this study appear to support the idea that “hands‐on” learning is more effective than verbal instruction for fostering retention and recall of the steps or the procedures of a skill in children.

Furthermore a contemporary theoretical framework called the dual‐process model (Van Lippevelde et al., 2016) was recently developed for interventions that improve children's physical health at school. The model draws on various theories and other models, including the elaboration likelihood model (Petty & Cacioppo, 1986) and social cognitive theory (Bandura, 1986). Behaviour change is promoted through an automatic pathway to target habits and a reflective pathway to target knowledge, attitude and self‐efficacy. Methods include the provision of rewards and positive reinforcement for habits, active learning and advance organisers for knowledge, and mere exposure and positive reinforcement for attitude and goal‐setting, monitoring and feedback for self‐efficacy.

### 1.4 Why it is important to do this review

Research in this area has to date not received much attention. As Dugan (2018) states and as already stated above “As most modern school systems require children to assume sedentary positions for extended periods of time, and the increasing use of classroom‐based technology adds to the number of hours seated. With the incidence of musculoskeletal pain reported not only in adult populations but increasingly in young people” knowing whether back health educational programmes are effective on spinal health, ergonomics and posture is a very important consideration for all stakeholders: pupils, teachers, parents, researchers as well as clinicians. Dugan's (2018) very recent systematic review on the diverse range of posture interventions used within primary schools suggests that “although approaches to promoting postural health in primary schools vary, studies could be compared in terms of their impetus e.g., low back pain in students, increasing classroom technology as well as aims and methodologies. Trends in the literature included delivery by posture experts (as opposed to homeroom teachers), examination of both sitting and moving postures in children and the impact of computer use on musculoskeletal heath. Much of the literature however relied largely on self‐report data and assessment instruments were wide‐ranging”.

Steele et al. (2006) in another systematic review evaluating the effectiveness of school‐based interventions on spinal pain was published more than 12 years ago. Twelve papers were included in this review with all papers receiving a “weak” quality rating. The result of this systematic review indicated that educational school‐based back health interventions may be effective in increasing spinal care knowledge and decreasing the prevalence of spinal pain. However, overall as the evidence was weak the results were inconclusive regarding spinal care behaviours. A more recent systematic review in a related area by Bonell et al. (2013) looked at other school‐based educational interventions but did not include educational interventions to improve back health knowledge and posture. Bonell et al. (2013) concluded however that whilst there is definitely the potential for school environment interventions to promote young people's health, the evidence base is far from definitive. Completing such an educational programme may help children improve their knowledge and develop an understanding of the importance of postural, ergonomic and spinal back‐health education for the prevention of back pain at a young age as well as later in adulthood. As stated previously the causes of LBP in youth are challenging and difficult to diagnose and have been theorised to include a lack of back care knowledge together with lack of knowledge regarding the best way of dealing with associated back problems. All the following: poor mechanical back shape, incorrect posture while using technology (game consoles, computers, Gameboys and iPhone), carrying heavy back packs, improper lifting techniques, incorrect posture during prolonged sitting and standing, lack of back care knowledge and the lack of opportunity to move around frequently (in standing, sitting) which may interfere with the educational environment. Inadequate or unavailable school lockers and ill‐fitting school furniture have also been implicated (Bettany‐Saltikov et al., 2008; Cardon et al., 2004; Feingold & Jacobs, 2002; Legg & Cruz, 2004; Sheldon, 1994). Therefore as research in this area is still controversial, a systematic review conducted with state of the art, high‐quality Campbell Collaboration methodology is urgently needed to update all stakeholders (parents, children, teachers, researchers and clinicians) with the latest evidence to help inform policy and practice in this area of public health.

## 2 OBJECTIVES

To assess the effectiveness of school‐based education programmes on back health for improving knowledge of back health, ergonomics and postural behaviour in school children aged 4–18 years.

## 3 METHODS

### 3.1 Criteria for considering studies for this review

#### 3.1.1 Types of studies

In the primary analysis, we will combine the results of randomised controlled trials (RCTs), Cluster RCTs and quasi‐randomised controlled trials. If a study is a well‐controlled RCT, then the experimental groups are assumed equal at the start of the study. We will also include prospective nonrandomised studies (NRSs) with a control group because it is anticipated that very few RCTs will be found. These will also include controlled before‐after studies and interrupted time series studies. Only controlled studies that are nonrandomised will require a pre‐ and post‐test. The studies will need to include, at least, a treatment and a control group. The studies may be written in English, Spanish, French, Italian, Maltese, Dutch and Indian. Retrospective studies as well as qualitative studies will be excluded. Narrative and other types of nonsystematic reviews (e.g., critical reviews, overviews, state‐of‐the‐art reviews), clinical practice guidelines, evidence summaries, critically appraised topics, clinical paths, consumer information sheets, best practice information sheets, technical reports and other evidence‐based pieces, will be excluded.

#### 3.1.2 Types of participants

We will include all children and young people between 4 and 18 years of age, attending school. Exclusion criteria: children under 4 years of age and adults over 18 years of age; chronic disease or conditions or comorbidities. Studies in which all subjects in the sample present with pain, spinal diseases or surgical vertebral treatment will be excluded.

#### 3.1.3 Types of interventions

The intervention of interest in this systematic review will be any formal educational school‐based programme that includes back health, ergonomics and postural behaviour that is designed to support the academic success of students' knowledge of posture and ergonomics within an educational establishment**.** Educational establishments, or schools are broadly defined as institutions dedicated to education. To be eligible, the interventions must engage school children in some form of active learning that cognitively and physically engages them in learning to improve their knowledge of ergonomics and postural behaviour. The contents (lectures or lectures with actual demonstrations and practice, workshops, individual lessons, class group lessons, curriculum lessons, educational modules), length (hours, days, weeks, months and years) and manner of delivery (face to face, face to face with complementary materials, group and individual practical participation, observations) of the programme may vary in each of the studies to be included as there is no standard school‐based educational programme for improving knowledge of back health, ergonomics and postural behaviour of school children. Physical activity or exercise only interventions will be excluded.

The control condition will include “usual” health and physical education programmes provided by schools or no educational school programme on back health and posture interventions. Studies comparing the effects of back health education programmes to another type of back health intervention, will not be included.

#### 3.1.4 Types of outcome measures

To the researchers' knowledge there is no consensus regarding indicators for outcome measurement in the evaluation of educational and health promotion programmes. While a change in a health outcome is the overall goal of most programmes, often such changes may not occur within the evaluation timeframe, and intermediate endpoints must also be measured to gauge effectiveness. Intermediate endpoints often evaluated after the implementation of a health promotion programme are the level of knowledge regarding the health issue, and the frequency in which relevant health behaviours are undertaken. The construction and administration of the outcome measures are typically developed by primary study authors. There may however be the occassional paper where these measures are both standardised and validated. Most measures of self‐report appear to be undertaken by the youth themselves.

### 3.2 Primary outcomes

Studies will be included that examine at least one of the following outcome measures:

1. Backcare knowledge
2. Knowledge of back care ergonomics
3. Back care behaviours
4. Knowledge of back posture

All outcomes (primary and secondary) will be measured at the beginning^1^ ^1^ and the end of the educational programme (weeks) and longer term (months, years). Any outcomes not mentioned above that are related to postural behavioural change will also be included. The included studies will include validated outcome measures that relate to the knowledge and/or understanding of all of the above using the results of surveys, actual measurements and other validated specific questionnaires.

### 3.3 Secondary outcomes

Secondary outcomes which include any adverse effects, for example pain or stiffness or other adverse effects reported in the included studies. If adverse effects are reported that are not listed here, we will still report them in our review.

#### 3.3.1 Duration of follow‐up

We will evaluate any pupils followed up for 6 months, 1 and 2 years.

#### 3.3.2 Types of settings

We will include any school setting and exclude any studies undertaken at a university (>18 years of age) level or any kindergarten educational setting that teaches children aged under 4 years.

## 4 SEARCH METHODS FOR IDENTIFICATION OF STUDIES

### 4.1 Search strategy

#### 4.1.1 Electronic searches

We will develop a comprehensive search strategy consisting of relevant terms and search electronic databases (MEDLINE, Embase, ERIC, Cumulative Index to Nursing and Allied Health Literature (CINAHL), Cochrane Central Register of Controlled Trials (CENTRAL), Scopus, Best Evidence Medical Education, Web of Knowledge, Google Scholar, PsycInfo) and dissertation databases (ProQuest) for relevant studies. We will not apply any language restrictions. We will search the following electronic databases and propose to search the literature since 1980. Below are some examples of the various databases which will be accessed. The Campbell (C2) Library (1980 to present) CINAHL (1980 to present). The Cochrane library, Health Management Information Consortium (1980 to present). ERIC (1980 to present). Europe PubMed Central (1980 to present). Australian Educational Index (1980 to present). British Educational Index (1980 to present). CAB Health (1980 to present).

#### 4.1.2 Searching other resources

We will complement our search with a thorough examination of reference lists of identified studies and will contact experts in the field to identify any ongoing or unpublished studies. We will also search trial registries (ICTRP) for ongoing studies. The following strategies will also be used: screening the reference lists of all relevant papers; searching the main electronic sources of ongoing trials. Searching the grey literature, including conference proceedings and Ph.D. theses completed since 1980. Contacting investigators and authors in this field for information on unpublished or incomplete trials. All searches will include non‐English language literature.

The search strategy aims to find both published and unpublished studies. A three‐step search strategy will be utilised in this review. An initial limited search of MEDLINE and CINAHL will be undertaken followed by analysis of the text words contained in the title and abstract, and of the index terms used to describe the article. A second search using all identified keywords and index terms will then be undertaken across all included databases. Third, the reference list of all identified reports and articles will be searched for additional studies. Studies published in English will be considered for inclusion in this review. There will be no restriction by date of publication.

The databases to be searched include:

● MEDLINE using EBSCO*host.*
● The CINAHL, using EBSCO*host*.
● Allied and Complementary MEDicine (AMED), using EBSCO*host.*
● EMBASE, using Ovid Online.
● Health Technology Assessment Database (HTA).
● Physiotherapy Evidence Database (PEDro).
● ProQuest Nursing and Allied Health Source.
● Scopus.
● SportDISCUS, using EBSCO*host.*
● Web of Science.
● ZETOC.
● The CENTRAL in *The Cochrane Library.*
● European Spine Journal.
● Journal of Bodywork and Movement Therapies.
● Physical Therapy in Sport.
● Physiotherapy.
● Spine.

An example of the CINAHL search strategy has been included in Appendices 1.

### 4.2 Data collection and analysis

#### 4.2.1 Selection of studies

A data selection form will first be developed on the basis of the inclusion criteria and will then be piloted and tested for both intraobserver and interobserver reliability by two review authors, who will then independently screen the search results by reading titles and abstracts. Potentially relevant studies will be obtained in full text and once again they will be independently assessed for inclusion by two review authors, who will resolve disagreement through discussion. A third review author will be contacted if disagreements persist. If a review author is also the author of a paper, another review author who has not authored any of the papers will undertake the selection.

#### 4.2.2 Data extraction and management

Two authors will independently screen the search outputs and abstracts for relevant studies. Full texts of studies with seemingly relevant abstracts will be retrieved and assessed for eligibility using the prespecified inclusion criteria. Studies will be classified as either included, excluded, awaiting assessment, or ongoing. Two authors will independently extract data from relevant studies. In the case of differences in the extracted data, we will discuss these to reach consensus, and if unresolved, these will be discussed with a third author. In the case of missing data, we will contact the original study author for clarification.

Data on the following will be extracted from included studies:

##### Study design

● Type of study
● Duration of study
● Country where study was conducted

##### Participants

● Number of participants
● Type of participants
● Level of education

##### Interventions and control

● Theory underlying intervention: biomedical or biopsychosocial (the terms biomedical and biopsychosocial refers to models of health. The Biomedical model basically focuses on abnormal genetics or physiology or pathology as the cause of illness (essentially biological causes), while the biopsychosocial model emphasises the importance of biological and psychological functioning as well as the social environment. The biomedical model is good for simple diseases like an appendicitis or pneumonia. The biopsychosocial is a better model for complex illnesses like depression or chronic pain. For back problems both models are generally used).

##### Intervention design

● Educational content
● Duration
● Intensity
● Timing of intervention

##### Intervention delivery

● The educational programme will consist of any education programme that will include the anatomy and structure of the spine, ergonomic principles associated with any activities of school life, and principles of postural positioning associated with lifting, pushing, pulling and any other activities of school life.

#### 4.2.3 Outcomes

##### Primary and secondary outcomes

● Measurement details (e.g., definition of outcome, tools used to measure outcome)
● Time point at which outcomes were measured

*Any of these outcomes or similar outcomes will also be extracted*: some examples of specific coding features have been included. These are not necessarily the ones that will be used in the final review but they will be similar.

**Knowledge of:**

**Standards of school bag features**: in millimetres or kilos.

**Ideal strap length**: in mm and weight in kilos.

**Best ways of carrying the bag and heavy bags:** (a) with two straps on the back, (b) on one shoulder.

**Best way of moving a bench or work table**: (a) the table needs to be kept far from the body, (b) the table needs to be kept close to the body.

**Best ways of carrying an object**: (a) on one shoulder, (b) on the back and (c) on the front of the body.

**Ideal body posture when moving objects**: (a) with hips and knees at any angle and the back bent forward and (b) with the back straight and hips and knees bent

**Natural curvature of the spine**: (a) normal, (b) small, (c) medium and (d) large curvature.

**Best way of relaxing the back during break time**: (a) back straight, (b) back slouched and (c) back in any position that is comfortable.

**Best posture when sleeping**: (a) on the back, (b) on ones tummy and (c) sideways with knees bent.

**Best position to put feet position on the floor when sitting**: (a) with hips and knees at right angles and (b) with hips and knees at any angle

**Space between the back of the knees and the leading edge of the chair**: (a) the back of the knees must touch the chair and (b) the back of the knees need to be over 1 cm from the edge of the chair.

**Space between the top of the thighs and the underside of the desk**: (a) the top of the thighs and the underside of the desk need to be touching and (b) the the top of the thighs and the underside of the desk must not touch.

**appropriate desk height when sitting on the chair:**

**Behaviour section**: **Any of these outcomes or similar outcomes will be extracted:**

**Knowledge of the following behaviours:**

Best student school bag features.

Best sports activities during a week.

Best ways of relaxing the back during break time.

Best way of bending knees or back when lifting objects or tying shoes.

How close one needs to stand to an object when lifting.

Asking for help when lifting heavy objects.

Best way of carrying the school bag.

Daily checking of bag weight.

Placing book/homework on an inclined writing surface of desk/working table.

Using back rest when sitting in the chair, body posture when doing homework.

Body posture when sitting in the chair, placing books on the tablet arm of the chair.

A proposed data extraction form has been included in Appendices section. However this form is subject to change for the full review when all the papers have been assessed.

#### 4.2.4 Assessment of risk of bias in included studies

The risk of bias for both randomised studies and NRSs will be assessed using the criteria recommended by the Cochrane Back Review Group (Furlan, Pennick, Bombardier, & van Tulder, 2009; Higgins & Green, 2011), together with items from the Downs and Black (1998) checklist, as outlined in Appendix 2. These criteria fall into five bias categories: selection bias, performance bias, attrition bias, detection bias and selective outcome reporting. The “assessment of risk of bias” form will be piloted and tested for intraobserver and interobserver reliability. Two review authors will independently assess the internal validity of the included studies. Any disagreement between the review authors will be resolved by discussion; a third independent review author will be consulted if disagreements persist. Risk of bias assessment will be blinded to trial authors, institution and journal. The risk of bias criteria will be scored as high, low or unclear and will be reported in the “risk of bias” table. The overall extent of risk of bias within each bias category (e.g., performance bias) will then be rated as “Bias” or “No bias”. Whilst it is difficult to provide an exhaustive list of all possible confounding variables at the start of the review, the review authors have experience in this field and are aware of most of the potential confounding variables that may occur when different treatment groups are compared. These may include, for instance, demographic variables such as age, When it comes to grading the quality of the evidence, evidence from studies judged “no bias” for all five categories will not be downgraded. Evidence will be downgraded (−1 point) when three or fewer categories for each study are judged to have bias. Evidence will be downgraded by −2 points when four or more categories for each study are judged to have bias. See Appendices section for the detailed criteria.

##### Treatment of qualitative research

We do not plan to include qualitative research.

### 4.3 MEASURES OF TREATMENT EFFECT

#### 4.3.1 Effect size

##### Dichotomous data

Where outcomes are reported as dichotomous data, we will use odds ratios with a 95% confidence [VW1] interval (CI) to summarise results within each study.

##### Continuous data

Where outcomes on the same scale are presented, we will use a mean difference. If the scales used are different then a standardised mean difference Hedges et al. (2010) will be used to combine effects across studies. If possible, missing effect sizes will be computed using other statistics presented in reports (e.g., *p*‐values, standard errors, confidence intervals or *T*‐values) using the RevMan calculator (Review Manager (RevMan, 2014)). Missing SDs will be imputed using data from included studies as suggested by Higgins and Green (2011).

#### 4.3.2 Publication bias

Publication bias for published versus unpublished work will be conducted by visually reviewing funnel plots to investigate any relationship between effect size and SE, provided sufficient studies have been identified, that is, 10 studies or more. Where we identify such a relationship, we will use Egger's test to test for funnel plot asymmetry (Egger, Davey Smith, Schneider, & Minder, 1997).

#### 4.3.3 Unit of analysis issues

In cases where three or more interventions are evaluated in a single study, we will include each pair‐wise comparison separately.

#### 4.3.4 Dealing with missing data

For recent papers (within 5 years), we will endeavour to collect missing data by contacting the authors. When data are insufficient to be entered into the meta‐analysis (even after contacting the authors), we will report the results qualitatively in the “table of characteristics of Included studies” and in the “summary of findings tables”.

#### 4.3.5 Assessment of heterogeneity

Heterogeneity will be assessed by comparing factors such as pupil demographics, type of intervention, types of control conditions and outcome measures. Statistical heterogeneity will be analysed and reported using outputs from RevMan for overall and subgroup analysis. Statistical heterogeneity will be assessed visually and by examining the *I*² statistic, which describes the approximate proportion of variation that is due to heterogeneity rather than sampling error. This will be supplemented by the *χ* ^2^ test, where a *p* < 0.05 indicates heterogeneity of intervention effects. In addition, we will estimate and present *τ* ^2^, along with its CIs, as an estimate of the magnitude of variation between studies. This will provide an estimate of the amount of between‐study variation. Sensitivity‐ and meta‐regression analyses will also be used to investigate possible sources of heterogeneity (please see below).

#### 4.3.6 Assessment of reporting biases

We will assess reporting biases to determine whether publication bias is present and we will construct funnel plots when at least 10 studies are available for the meta‐analysis (Sutton et al., 2000).

#### 4.3.7 Data synthesis

Summary and descriptive statistics of the study‐level characteristics, methodological quality characteristics, and participant and intervention characteristics will be tabulated to describe the included studies. Due to the anticipated between study variability, a random effects model will be used throughout the analysis using the inverse variance estimation method (Borenstein et al., 2011). Analysis will be carried out using RevMan and CMA software. Meta‐regression analyses will be performed using CMA software to explore heterogeneity between subgroups based on age, gender and country of origin. Data from some studies may be published in multiple reports so care will be taken to identify these nonindependent results. If more than one article reports study findings that were all based on the same sample, all the different reports may contribute information to the coding manual. Multiple publications will be identified by finding characteristics such as identical sample sizes, authors, intervention programmes or outcome reports. Because multiple publications can lead to an incorrect weighting of study results, authors will be contacted if there are uncertainties regarding the multiple publication of original research.

Where a study includes more than one treatment arm compared with a control group (if enough studies are found to allow for this) we will conduct separate meta‐analyses for each treatment arm. If not, we will combine effect sizes to create a single pair‐wise comparison (Higgins & Green, 2011). For dichotomous data, we will sum the sample sizes and events across groups. For continuous data, we will combine sample sizes, means and SDs according to the formula detailed in the Cochrane Handbook for Systematic Reviews of Interventions (Higgins & Green, 2011). To account for statistical dependencies robust variance estimation will be used (Hedges et al., 2010). If studies report multiple measures of the same construct at different points in time, we will conduct separate meta‐analyses for outcomes measured at several periods of follow‐up: before the intervention, 4–6 weeks after the intervention, and possibly at 3 months, 6 months and 1 year or 2 years after the intervention, if such data are available. If, within any of these periods, the included studies report measures more than once, then we will obtain a single summary effect within that time period.

#### 4.3.8 Subgroup analysis and investigation of heterogeneity

Subgroup analysis will be assessed through the comparison of the following: participant demographics (age 4–10 vs. 11–14 vs. 15–18 years) social class (private vs. public schools), type of intervention, (practical vs. nonpractical) and length of intervention (short duration [hours or days] vs. long duration [weeks and months]). Conducting these subgroup analysis will establish the generalisability of the effect of the education programmes by age, social class, type of intervention and length of intervention, respectively.

#### 4.3.9 Sensitivity analysis

Sensitivity analysis will be conducted to determine whether the overall results of data analysis are influenced by removal of:

- Unpublished studies
- Studies with outlier effect sizes
- Studies with high risk of bias
- Studies with missing information (e.g., incomplete presentation of finding)

## 5 RESULTS

### 5.1 Description of studies

**Results of the search**

**Included studies**

**Excluded studies**

### 5.2 Risk of bias in included studies

**Allocation (selection bias)**

**Blinding (performance bias and detection bias)**

**Incomplete outcome data (attrition bias)**

**Selective reporting (reporting bias)**

**Other potential sources of bias**

**Effects of interventions**

## 6 DISCUSSION

**Summary of main results**

**Overall completeness and applicability of evidence**

**Quality of the evidence**

**Potential biases in the review process**

**Agreements and disagreements with other studies or reviews**

## 7 AUTHORS' CONCLUSIONS

**Implications for practice**

**Implications for research**

## AUTHOR CONTRIBUTIONS

Substantial contributions to conception and design: Josette Bettany‐Saltikov, Robert Mcsherry, Tracey Arnell, Paul Van Schaik

Study search and selection: Julie Hogg, Josette Bettany‐Saltikov, Gok Kandasamy, Paul Van Schaik

Methodological assessment: Josette Bettany‐Saltikov, Gok Kandasamy, Garikotz Aristegui, Tracey Arnell, Robert Mcsherry, Paul Van Schaik

Acquisition/abstraction of data: Josette Bettany‐Saltikov, Gok Kandasamy, Garikotz Aristegui, Tracey Arnell, Robert Mcsherry, Paul Van Schaik

Data analysis: Victoria Whittaker, Josette Bettany‐Saltikov.Gok Kandasamy

Interpretation of data: Victoria Whittaker, Josette Bettany‐Saltikov, Gok Kandasamy, Garikotz Aristegui, Tracey Arnell, Robert Mcsherry, Paul Van Schaik

Drafting of the article: Josette Bettany‐Saltikov, Gok Kandasamy, Garikotz Aristegui, Tracey Arnell, Robert Mcsherry, Paul Van Schaik

Critical revision for important intellectual content: Josette Bettany‐Saltikov, Paul Van Schaik, Robert Mcsherry.

## DECLARATIONS OF INTEREST

We have no conflicts of interests.

## DIFFERENCES BETWEEN PROTOCOL AND REVIEW

### Published notes

**Characteristics of studies**

**Characteristics of included studies**

**Characteristics of excluded studies**

**Characteristics of studies awaiting classification**

**Characteristics of ongoing studies**

**Summary of findings tables**

**Additional tables**

**References to studies**

**Included studies**

**Excluded studies**

**Studies awaiting classification**

**Ongoing studies**

**Other references**

**Classification pending references**

**Data and analyses**

**Sources of support**

**Internal sources**

- No sources of support provided

**External sources**

- No sources of support provided

## Supporting information

Supporting information

## Footnotes

1 COMMENT: Only if there is a pretest: RCTs with no pretest are not eligible for inclusion.
