# Supplementary material for: PROTOCOL: School‐based education programmes for improving knowledge of back health, ergonomics and postural behaviour of school children aged 4–18: A systematic review
Source: Campbell Syst Rev. 2019 Jul 13;15(1-2):e1014. doi: 10.1002/cl2.1014 (PMC8356490; doi:10.1002/cl2.1014)
Supplement: Supplementary file 1 — Supporting information [file CL2-15-e1014-s001.docx]

**Appendices**

**1 Cinahl search strategy**

**School-based education programs for improving knowledge of back health, ergonomics and postural behaviour of school children**

**CINAHL Search Strategy**

S1 child*

S2 (MH "Child▒+▒")

S3 (MH "Adolescence")

S4 adolescen*

S5 teen*

S6 youth*

S7 young*

S8 student*

S9 pupil*

S10 S1 OR S2 OR S3 OR S4 OR S5 OR S6 OR S7 OR S8 OR S9

S11 (MH "Students, High School")

S12 (MH "Students, Middle School")

S13 (MH "Schools, Secondary")

S14 secondary school

S15 high school

S16 highschool

S17 (MH "Schools, Middle")

S18 middle school

S19 ("6th grade" OR "grade 6" OR "sixth grade" OR "sixth-grade" OR

"7th grade" OR "grade 7" OR "seventh grade" OR "seventh-grade"

OR "8th grade" OR "grade 8 OR "eighth grade" OR "9th grade" OR

"grade 9" OR "ninth grade" OR "10th grade" OR "grade 10" OR

"tenth grade" OR "11th grade" OR "grade 11" OR "eleventh grade"

OR "12th grade" OR "grade 12" OR "twelfth grade")

S20 S11 OR S12 OR S13 OR S14 OR S15 OR S16 OR S17 OR

S18 OR S19

S21 S10 AND S20

S22 (MH "School Health Services")

S23 (MH "School Health Education")

S24 (MH "Child Safety/ED")

S25 "safety education"

S26 (MH "Preventive Health Care")

S27 primary prevention

S28 (MH "Health Promotion")

S29 (MH "Health Education")

S30 S24 OR S25 OR S26 OR S27 OR S28 OR S29

S31 school*

S32 S30 AND S31

S33 (school* OR classroom*) N9 (education OR promotion OR intervention*

OR initiative* OR program* OR presentation* OR lesson* OR

campaign* OR instruction*)

S34 S22 OR S23 OR S32 OR S33

S35 (MH "Health Knowledge")

S36 (MH "Attitude to Health")

S37 (MH "Health Beliefs")

S38 (MH "Attitude to Risk")

S39 (MH "Risk Taking Behavior")

S40 (MH "Behavioral Changes")

S41 (MH "Health Behavior")

S42 S35 OR S36 OR S37 OR S38 OR S39 OR S40 OR S41

S43 (MH "Back Pain▒+▒")

S44 (MH "Back Injuries▒+▒")

S45 (MH "Spinal Cord Injuries▒+▒")

S46 (MH "Ergonomics")

S47 (MH "Posture")

S48 S43 OR S44 OR S45 OR S46 OR S47

S49 S42 AND S48

S50 (knowledge OR attitude* OR awareness OR information level* OR

health belief* OR safety belief* OR lifestyle OR life-style OR

health behavio* OR risk* OR prevent*) N9 (postur* OR ergonomics

OR back health OR spin* health OR back pain OR spin* pain OR

back care OR spin* care OR back injur* OR spin* injur* OR

back problem* OR spin* problem*)

S51 S49 OR S50

S52 S21 AND S34 AND S51

**2 Appendix 3. Criteria for assessing risk of bias for internal validity for randomised and nonrandomised studies (Downs and Black 1998; Furlan 2009)**

Selection **bias**

**Random sequence generation**

Risk of selection bias is low if the investigators describe a random component in the sequence generation process, such as referring to a random number table, using a computer random number generator, coin tossing, shuffling cards or envelopes, throwing dice,drawing lots, minimising (minimisation may be implemented without a random element, and this is considered to be equivalent to being random). Risk of selection bias is high if the investigators describe a non-random component in the sequence generation process, such as sequence generated by odd or even date of birth, date (or day) of admission, hospital or clinic record number or allocation by judgement of the clinician, preference of the participant, results of a laboratory test or a series of tests or availability of the intervention. If it is a non-randomised study, this will be rated as high bias.

**Allocation concealment** Risk of selection bias is low if participants and investigators enrolling participants could not foresee assignment because one of the following, or an equivalent method, was used to conceal allocation: central allocation (including telephone,Web-based and pharmacycontrolled randomisation); sequentially numbered drug containers of identical appearance; or sequentially numbered, opaque, sealed envelopes. Risk of bias is high if participants or investigators enrolling participants could possibly foresee assignments and thus introduce selection bias, such as allocation based on using an open random allocation schedule (e.g. a list of random numbers); assignment envelopes were used without appropriate safeguards (e.g. if envelopes were unsealed or non-opaque or were not sequentially numbered); alternation or rotation; date of birth; case record number; or other explicitly unconcealed procedures. If it is a non-randomised study, this will be rated as high bias.

**Selection bias (population)*** Risk of selection bias is low if participants in different intervention groups are recruited from the same population. Selection bias (timing)* Risk of selection bias is low if participants in different intervention groups are recruited over the same time. Surgical studies must be▒<▒10 years old for low risk of selection bias.

**Adjustment for confounding*** Risk is low if no significant group differences were shown. Risk is high if the effect of the main confounders was not investigated or if no adjustment was made in the final analyses. **Performance bias**

**Blinding of participants** Risk of performance bias is low if blinding of participants was ensured and it was unlikely that the blinding could have been broken; or if no blinding or incomplete blinding was performed, but the review authors judge that the outcome is not likely to be influenced by lack of blinding.

**Blinding of personnel/ care providers** Risk of performance bias is low if blinding of personnel was ensured and it was unlikely that the blinding could have been broken; or if no blinding or incomplete blinding was performed, but the review authors judge that the outcome is not likely to be influenced by lack of blinding.

**Compliance (adherence)** Risk of bias is low if compliance with the interventions was acceptable on the basis of reported intensity/dosage, duration, number and frequency for both index and control intervention(s). For single-session interventions (e.g. surgery), this item is irrelevant.

**Cointerventions** Risk of bias is low if no co interventions were provided, or if they were similar between index and control groups.

**Attrition bias Incomplete outcome data** Risk of attrition bias is low if no outcome data were missing; reasons for missing outcome data were unlikely to be related to the true outcome (for survival data, censoring unlikely to be introducing bias); missing outcome data were balanced in numbers, with similar reasons for missing data across groups; for dichotomous outcome data, the proportion ofmissing outcomes compared with the observed event risk was not enough to have a clinically relevant impact on the intervention effect estimate; for continuous outcome data, the plausible effect size (difference in means or standardised difference in means) among missing outcomes was not enough to have a clinically relevant impact on observed effect size, or missing data were imputed using appropriate methods (if dropouts are very large, imputation using even “acceptable” methods may still suggest a high risk of bias). The percentage of withdrawals and dropouts should not exceed 20% for short-term follow-up and 30% for long-term follow-up and should not lead to substantial bias (these percentages are commonly used but arbitrary and are not supported by the literature).

**Intention-to-treat analysis** Risk of bias is low if all randomly assigned participants were reported/analysed in the group to which they were allocated by randomisation. Measurement/detection

**Blinding of outcome assessment** Risk of detection bias is low if blinding of the outcome assessment was ensured and it was unlikely that the blinding could have been broken; or if no blinding or incomplete blinding was performed, but the review authors judge that the outcome is not likely to be influenced by lack of blinding, or: • for participant-reported outcomes in which the participant was the outcome assessor (e.g. pain, disability): Risk of bias for outcome assessors is low if risk of bias for participant blinding is low; • for outcome criteria that are clinical or therapeutic events that will be determined by the interaction between participants and care providers (e.g. co interventions, length of hospitalisation, treatment failure), in which the care provider is the outcome assessor: Risk of bias for outcome assessors is low if risk of bias for care providers is low; and • for outcome criteria that are assessed from data from medical forms: Risk of bias is low if the treatment or adverse effects of the treatment could not be noticed in the extracted data.

**Timing of outcome assessments** Risk of bias is low if all important outcome assessments for all intervention groups were measured at the same time, or if analyses adjust for different lengths of follow-up. Selective reporting

**Data dredging** Risk of bias is low if all analyses were planned at the outset of the study. Surgical versus non-surgical interventions in patients with adolescent idiopathic scoliosis (Protocol) 19 Copyright © 2013 The Cochrane Collaboration. Published by JohnWiley & Sons, Ltd. Risk of bias is high if analyses were conducted retrospectively (e.g. retrospective unplanned subgroup analyses).

**Outcome measures** Risk of reporting bias is low if the study protocol is available and all of the study’s prespecified (primary and secondary) outcomes that are of interest in the review have been reported in the prespecified way, or if the study protocol is not available, but it is clear that the published reports include all expected outcomes, including those that were prespecified (convincing text of this nature may be uncommon).

Risk of reporting bias is high if not all of the study’s prespecified primary outcomes have been reported; one or more primary outcomes are reported using measurements, analysis methods or subsets of the data (e.g. sub scales) that were not prespecified; one or more reported primary outcomes were not prespecified (unless clear justification for their reporting is provided, such as an unexpected adverse effect); one or more outcomes of interest in the review are reported incompletely, so that they cannot be entered into a meta-analysis; the study report fails to include results for a key outcome that would be expected to have been reported for such a study. *Items are relevant only to non-randomised studies.

**3 Proposed data extraction form**

| **Data extraction table** | |  |
| --- | --- | --- |
| **Date of data extraction:** |  | additional comments/details |
| **Reviewer:** |  |  |
| **Bibliographical details of study:** |  |  |
| **Purpose of study:** |  |  |
| **Study design:** |  |  |
| o Type of study |  |  |
| o Duration of study |  |  |
| o Country where study was conducted |  |  |
| **Setting:** |  |  |
| **Population:** |  |  |
| **Age:** |  |  |
| **Education, school year:** |  |  |
| **Gender:** No of boys No of girls |  |  |
| **Intervention Group** |  |  |
| **Numbers In intervention group** |  |  |
| **Theory underlying intervention** |  |  |
| **Intervention design** |  |  |
| **Educational content** |  |  |
| **intensity** |  |  |
| **timing of intervention** |  |  |
| **Duration** |  |  |
| **Intervention delivery: Yes/No/alternative** | |  |
| The educational program will consist of any education program, that include the | |  |
| anatomy and structure of the spine, ergonomic principles associated with any activities of school life, | |  |
| principles of postural positioning associated with lifting, pushing, pulling and any other activities of school life. | |  |
| **Comparative intervention:** |  | Additional comments/ details |
| **Total Numbers** |  |  |
| **No of boys and no of girls** |  |  |
| **Theory underlying comparative intervention**: |  |  |
| **Intervention design** |  |  |
| **Educational content** |  |  |
| **intensity** |  |  |
| **Timing of intervention** |  |  |
| **Duration** |  |  |
| **Comparative Intervention delivery** |  |  |
| **Will consist of usual physical activity and Physical education exercises** |  |  |
| Differs from intervention in other ways (please describe) |  |  |
| **Outcomes** | **yes/no/unclear** | Additional comments/ details |
| Measurement details (e.g. definition of outcome,) |  |  |
| tools used to measure outcome |  |  |
| Time point at which outcomes were measured |  |  |
| natural curvature of the spine |  |  |
| Knowledge of best ways of carrying the bag and heavy bags |  |  |
| Knowledge of best way of moving a bench or work table, |  |  |
| Knowledge of best ways of carrying an object |  |  |
| Knowledge of body posture when moving objects |  |  |
| Knowledge of best position to put feet position on the floor |  |  |
| Knowledge of best posture when sleeping |  |  |
| Knowledge of space between the top of the thighs and the underside of the desk, appropriate desk height when sitting on the chair. |  |  |
| **Behaviour section: Any of these outcomes or similar outcomes will be extracted** |  |  |
| Knowledge of best student school bag features, |  |  |
| Knowledge of best sports activities during a week, |  |  |
| Knowledge of ways of relaxing the back during break time |  |  |
| Knowledge of best way of bending knees or back when lifting objects or tying shoes, |  |  |
| Knowledge of how close one needs to stand to an object when lifting, |  |  |
| Knowledge of Asking for help when lifting heavy objects, |  |  |
| Knowledge of best way of carrying the school bag, |  |  |
| Knowledge of daily checking of bag weight |  |  |
| Knowledge of placing book/homework on an inclined writing surface of desk/working table, |  |  |
| Knowledge of using back rest when sitting in the chair, |  |  |
| Knowledge of better body postures when doing homework, |  |  |
| Knowledge of better body postures when sitting in the chair, |  |  |
| Knowledge of how to place books on the tablet arm of the chair. |  |  |
